# The Small GTPase Rheb Affects Central Brain Neuronal Morphology and Memory Formation in *Drosophila*

**DOI:** 10.1371/journal.pone.0044888

**Published:** 2012-09-19

**Authors:** Heather L. D. Brown, Karla R. Kaun, Bruce A. Edgar

**Affiliations:** 1 Basic Sciences Division, Fred Hutchinson Cancer Research Center, Seattle, Washington, United States of America; 2 Department of Anatomy, University of California San Francisco, San Francisco, California, United States of America; National Cancer Institute, United States of America

## Abstract

Mutations in either of two tumor suppressor genes, *TSC1* or *TSC2*, cause tuberous sclerosis complex (TSC), a syndrome resulting in benign hamartomatous tumors and neurological disorders. Cellular growth defects and neuronal disorganization associated with TSC are believed to be due to upregulated TOR signaling. We overexpressed Rheb, an upstream regulator of TOR, in two different subsets of *D. melanogaster* central brain neurons in order to upregulate the Tsc-Rheb-TOR pathway. Overexpression of Rheb in either the mushroom bodies or the insulin producing cells resulted in enlarged axon projections and cell bodies, which continued to increase in size with prolonged Rheb expression as the animals aged. Additionally, Rheb overexpression in the mushroom bodies resulted in deficiencies in 3 hr but not immediate appetitive memory. Thus, Rheb overexpression in the central brain neurons of flies causes not only morphological phenotypes, but behavioral and aging phenotypes that may mirror symptoms of TSC.

## Introduction

Tuberous sclerosis complex (TSC) is a multisystem autosomal-dominant syndrome caused by mutations inactivating one of two tumor suppressor genes, *TSC1* or *TSC2*. It is typified by formation of hamartomas, or benign tumors, in multiple organ systems such as the heart, lungs, kidneys, brain and skin [1] and is also commonly connected with a wide array of potentially devastating neurological phenotypes, including intellectual, behavioral and psychiatric disorders. Some of the symptoms associated with TSC are directly attributed to the formation of brain hamartomas, also known as cortical tubers [2]. However, many of TSC's neurological manifestations, such as autism and cognitive impairment, are developmental in origin and may reflect a disorganization of portions of the central nervous system rather than disruption via cortical tubers [2], [3]. The developmental mechanisms disrupted in TSC are still being elucidated.

TSC1 and TSC2 form an obligate heterodimeric protein complex that functions downstream of PI3K/Akt signaling, and is a key regulator of the serine-threonine kinase TOR (Target of Rapamycin) [4], [5]. TSC2 contains a GAP (GTPase activating protein) domain in its c-terminal region, necessary for its function in inhibiting the activity of the small GTPase, Rheb [6]–[8]. Rheb, a homolog of Ras, activates TOR by binding to its kinase domain [9]. Through phosphorylation of ribosomal S6 kinase (S6K), the translational repressor 4E-BP1, and other factors, TOR activation upregulates translation and promotes cell growth [2], [4]. TOR has also been shown to be a potent suppressor of autophagy [10]–[12]. Loss of TSC1 or TSC2 function or experimental overexpression of Rheb results in upregulated TOR activity, as seen both by increased phosphorylated S6K and enhanced cellular growth that can be repressed through administration of rapamycin [6], [13]–[15].

Misregulation of the TOR pathway has been shown to have multiple effects on the neurons and glia of the central nervous system. In addition to disruption of proper brain function through formation of cortical tubers, symptoms of TSC have also been associated with other physiological and developmental malfunctions [3]. Loss of Tsc1 in the pyramidal neurons of mice causes an increase in the size of somata and dendritic spines [13], while mice lacking Tsc1 in all neurons have enlarged cortical and hippocampal neurons akin to tubers, as well as abnormal brain architecture, delays in myelination and seizures [16]. Additionally, astrocyte-specific *Tsc1* knockout mice have an increase in astrocyte numbers and abnormal organization of hippocampal neurons [17]. Mice heterozygous for loss-of-function of either *Tsc1* or *Tsc2* in neurons lack apparent neural morphological defects, but do display cognitive and social defects [18]–[20]. Taken together, the murine data alone shows a surprising phenotypic variability when TSC is modeled within the central nervous system.

The Tsc-Rheb-TOR pathway is highly conserved, and as such can be studied effectively in invertebrates such as the fruit fly, *Drosophila melanogaster*. *Rheb* was originally discovered in a *Drosophila* screen for novel regulators of cell growth [7], [8]; indeed, all of the major genetic players that comprise the human Tsc-Rheb-TOR pathway are present in flies. Overexpression of Rheb in *Drosophila* photoreceptor cells has been shown to produce axon guidance defects and induce cell death by downregulating autophagy [10], [21], while Rheb overexpression in the external sensory organ produces a cell fate switch from neuron to bristle/socket cells [22]. To investigate the effects of Rheb overexpression specifically within the *D. melanogaster* central brain, we used the *Gal4-UAS* system [23] to target two neuronal subsets, the mushroom bodies and insulin producing cells (IPCs). Rheb overexpression within marked central neurons (mushroom bodies) of a living animal allowed us to test behavioral output and investigate overall morphology, and compare our results from a *Drosophila* model to mammalian models of TSC.

## Methods

### Drosophila strains

To generate flies overexpressing both Rheb and GFP in the mushroom bodies, we crossed *y w mcd8GFP; +*, and *y w mcd8GFP; UAS-Rheb* (generated from Bloomington Stock Center stocks) to w*; OK107-Gal4, respectively. To generate flies overexpressing both Rheb and GFP in the insulin producing cells, we crossed w*; UAS-Rheb to w*; dilp2-Gal4.A UASmcd8-GFP/CyO. *w^1118^*, was crossed to w*; OK107-Gal4 and used for behavioral test controls. All stocks are available from Bloomington Stock Center, Indiana University.

Females were collected 1 day post-eclosion for immunohistochemistry. Males were collected 1 day post-eclosion for behavior tests. For aging experiments, 1 day old female adults were collected, then housed in food vials at 25°C for 21 days post-eclosion (PE); vials were changed every 3 to 4 days.

### Immunohistochemistry and Imaging

Drosophila brains were dissected in a phosphate-buffered saline solution, fixed for 30 minutes in 4% buffered paraformaldehyde, rinsed several times in phosphate-buffered saline with 0.3% Triton-X (PBS-Tx) and blocked with 5% normal goat serum for 15 minutes. After blocking, they were incubated overnight at 4°C in one or more of the following antibodies: mouse anti-fibrillarin (1∶500) [24], rabbit anti-GFP (1∶10,000; Invitrogen), and mouse anti-FasII 1D4 (1∶100; Developmental Studies Hybridoma Bank). They were then rinsed again in PBS-Tx, and incubated overnight at 4°C in secondary antibody (1∶1000; AlexaFluor 488 goat anti-rabbit IgG and AlexaFluor 568 goat anti-mouse IgG; Invitrogen) and DAPI (0.1 µg/mL; Invitrogen). Following secondary incubation, tissues were washed in PBS, attached to polylysine-coated coverslips, dehydrated through an ethanol series, cleared in xylene and mounted in DPX (Fluka BioChemica). All tissues were imaged on a Zeiss LSM 510 META confocal microscope.

### EdU Labeling

Staged embryos were collected on grape agar plates and either transferred to a food vial containing 0.02 mM Click-it™ EdU or allowed to hatch and pupate, at which time the newly eclosed adults were transferred to a food vial containing EdU. Larval animals were kept on EdU food until pupation, and were dissected at 1 day PE. Adults that were kept on EdU-containing food for 10 days were dissected on day 10 PE. Immunohistochemistry and imaging was performed as above, using the Click-it™ EdU Alexa Fluor-555 imaging kit from Invitrogen.

### Behavioral Tests

#### Odor sensitivity

Groups of 30 4–5 day old males food deprived for 16–20 hrs on agar vials prior to testing were tested. Odors used were 1∶36 ethyl acetate in mineral oil and 1∶36 iso-amyl alcohol in mineral oil. Choice tests were performed in a 1 cm diameter Y-maze with 13 cm arms, where flies chose between each single odor and air streamed through mineral oil. Preference index was calculated by (# flies in odor vial - # flies in air vial)/total # flies.

#### Sucrose sensitivity

Groups of 30 4–5 day old males food deprived for 16–20 hours on agar vials prior to testing were tested. Sensitivity chambers consisted of 6 cm Petri dishes with 2.1 cm diameter 3 MM filter paper folded along seam of dish on opposing sides. Filter paper was soaked in water or 2 M sucrose and dried 24 hours prior to experiment. Flies were gently tapped into the dish, and the number of flies on each filter paper was counted 30 seconds after entry. Preference index was calculated by (# flies on sucrose - # flies on water)/total # flies.

#### Sucrose Conditioning

Sucrose conditioning was performed similarly to Kaun et al [25]. Groups of 30 4–5 day old males food deprived for 16–20 hours on agar vials prior to testing were used. Flies were trained in 14 mL culture vials with mesh lids in 30×15×15 cm training boxes and tested in a 1 cm diameter Y-maze with 13 cm arms. Training consisted of a 5 minute habituation to the training chamber with air (flow rate 130), a 5 minute presentation of odor 1 with plain filter paper pre-soaked in water and dried, then 5 minutes of odor 2 with filter paper pre-soaked in 2 M sucrose and dried. Reciprocal training was performed to ensure that inherent preference for either odor did not affect conditioning scored: a separate group of flies was simultaneously trained using odor 1 as the sucrose-paired odor. Vials of flies from Group 1 and Group 2 were paired according to placement in the training chamber and tested simultaneously 2 minutes or 3 hours following training. Preference index was calculated by (# flies in Odor^+^ vial - # flies in Odor^−^ vial)/total # flies. Learning index was the average between preference indexes in reciprocal trials. Statistics for behavior experiments was performed using JMP 9.0.2. Statistical significance was determined using one-way ANOVA with two-way Student's-t post-hoc test (p<0.05).

### Image Analysis

Confocal image stacks were compiled and analyzed using ImageJ (http://rsb.info.nih.gov/ij/). Cell slice area and cluster volume for the Kenyon cells of the mushroom bodies and cell number for the IPCs were scored blind. For cell slice area, five cells per animal were measured. Volume analysis for both the cell cluster and neuropil volumes was performed using the ImageJ “Measure Stack” volume measurement plugin. IPC number was calculated manually by marking cell nuclei on each slice of an image stack, taking care to count each cell only once. Statistical significance was determined using one-way ANOVA with Tukey post-hoc test (p<0.05; SPSS 17.0, www.spss.com).

## Results

To determine if heightened expression of Rheb would affect development of the *D. melanogaster* nervous system and/or cause behavioral shifts, we overexpressed Rheb in a subset of neurons within the brain. We chose to use Rheb overexpression in the fly nervous system since it has previously been shown to upregulate TOR activity and gives similar phenotypes to *Tsc1* null clones [6], [20]–[22]; additionally, use of RNAi constructs seen to reduce Tsc1 or Tsc2 in other tissues were unsuccessful in these two neuronal subsets, presumably due to the resistance of the *Drosophila* nervous system to RNAi [26].

**Figure 1:**
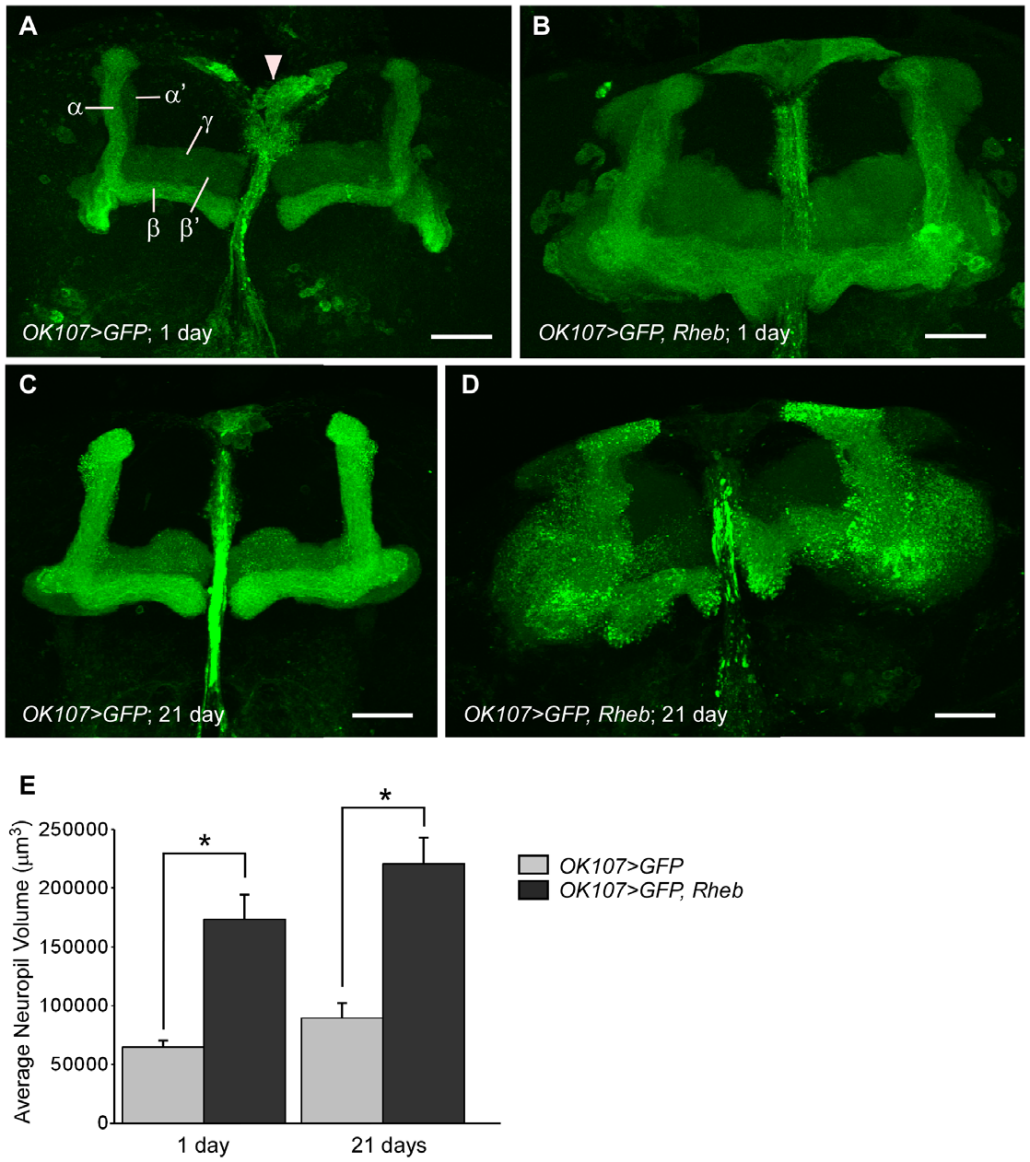
Overexpression of Rheb in the mushroom bodies results in enlarged axonal lobes. (A) A Z-stack projection of GAL4-driven membrane-bound GFP (CD8::GFP; green) in the mushroom body lobes of a 1 day post-eclosion (PE) fly using the *OK107* driver. The mushroom body is subdivided into α, α′, β, β′ and γ lobes. The *OK107* driver also produces expression in the nearby insulin producing cells (IPCs; arrowhead). (B) All lobes of a 1 day PE mushroom body expressing CD8::GFP and Rheb are expanded. (C) 21 days PE mushroom body lobes from control animals (CD8::GFP only). (D) Grossly enlarged 21 days PE mushroom body lobes expressing both CD8::GFP and Rheb. Scale bars are 30 µm. (E) Quantification of the average approximate neuropil volume, using the ImageJ Measure Stack volume plugin. Neuropil of neurons overexpressing Rheb are significantly larger than controls at both 1 and 21 days PE (N = 13–35, ANOVA, p = 0), but there is no significant difference between control groups of different ages (p>0.05 ) or between the two groups overexpressing Rheb (p>0.05). * indicates significance between bracketed columns. Error bars represent SE.

The mushroom bodies are an associative area within the insect brain and have been implicated in many complex behaviors, most notably in olfactory learning and memory [27]–[29]. In addition, the mushroom bodies are well-organized and characterized structures within the central brain, with a distinct developmental pattern. They are composed of neurons with bilaterally symmetrical axonal and dendritic projections. Within each *Drosophila* brain hemisphere, the mushroom body cell cluster is composed of approximately 2500 Kenyon cells. Each Kenyon cell extends dendritic projections to form the calyces, while the axonal projections continue their extensions to form two distinct branches, each subdivided into α, α′, β, β′, and γ lobes (Fig. 1A) [29]–[31]. We chose the mushroom bodies to test neuronal response to Rheb overexpression because of their distinct axonal structures and capacity for functional assessment by means of well-defined behavioral assays. Using the *Gal4/UAS* system [23], we targeted Rheb overexpression along with a membrane-bound GFP construct (CD8::GFP) to the mushroom body neurons using the *OK107-Gal4* driver [32]. CD8::GFP was highly expressed in both the cell bodies and neuropil of the mushroom bodies when driven by *OK107-Gal4*. In addition, *OK107-Gal4* also drives *UAS* transgene expression in the insulin producing cells (IPCs), a set of neurosecretory cells located along the midline of the brain (Fig. 1A, arrowhead), as well as at lower levels in a few other neurons within the brain [32]–[34]. However, the distinct structure of the mushroom bodies combined with *OK107*-driven expression in relatively few neurons outside of the mushroom bodies facilitated our analysis of mushroom body structures.

### Rheb overexpression in the *Drosophila* mushroom bodies

**Figure 2:**
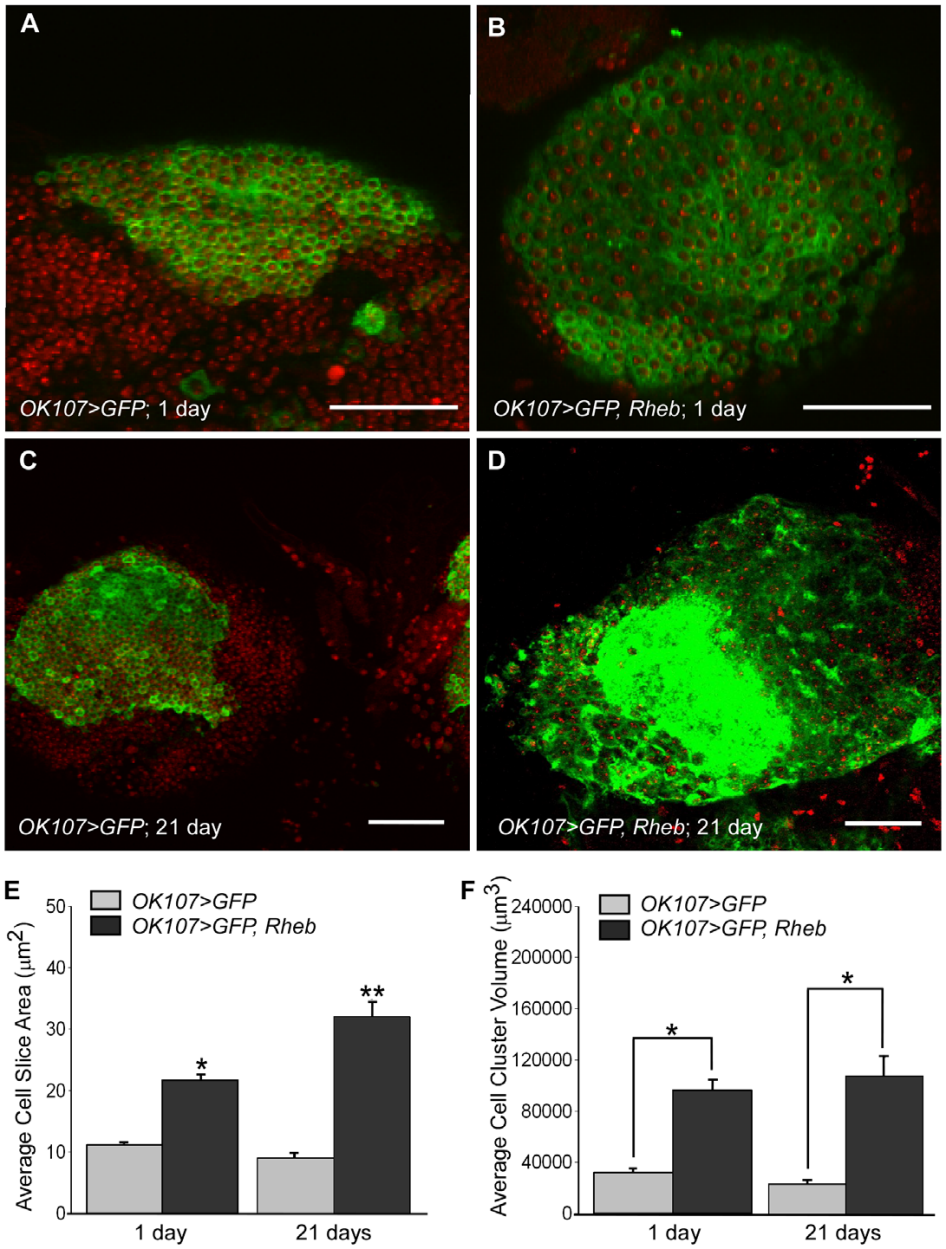
Rheb overexpression in the mushroom bodies causes enlargement of cell body size and cluster volume. (A) A single section through the Kenyon cell bodies of a 1 day PE animal expressing CD8::GFP only via the *OK107* driver; DNA is stained with DAPI (red). (B) Kenyon cell bodies of a 1 day PE animal overexpressing Rheb. The cells are larger, but the DAPI stained area appears similar to control animal cells. (C) Kenyon cell bodies at 21 days PE. (D) Enlarged Kenyon cell bodies overexpressing Rheb from a 21 days PE animal. Difficulty in distinguishing individual cells is due to diffuse localization of GFP combined with the greatly enlarged cell bodies. (E) Quantification of the average area of an optical slice through the center of a single Kenyon cell. Five cells per mushroom body sample were measured using ImageJ, then averaged. There is a statistically significant difference (p = 0) between all groups using one-way ANOVA, except between 1 day PE and 21 days PE control animals (N = 18–32, p>0.05). * and ** represent significant differences compared to all other columns. Error bars represent SE. (F) Quantification of the average approximate Kenyon cell cluster volume, using the ImageJ Measure Stack volume plugin. Cell clusters overexpressing Rheb are significantly larger than controls at both 1 and 21 days PE (N = 18–32, ANOVA, p = 0), but there is no significant difference between control groups of different ages (p>0.05 ) or between the two groups overexpressing Rheb (p>0.05). * indicates significance between bracketed columns. Error bars represent SE.

We evaluated the response to Rheb overexpression within the mushroom bodies by qualitative assessment of axonal lobe shape and size, as well as quantitative measurements of cell body size and cell cluster size. When examined at 1 day post-eclosion (PE), mushroom body axonal lobes overexpressing Rheb (*OK107>GFP, Rheb*) were greatly expanded as compared to the mushroom bodies of control animals (*OK107>GFP*; Fig. 1B). All portions of the lobe appeared enlarged. Neuropil volume was significantly larger for *OK107>GFP, Rheb* animals (173517.6 µm^3±^20829.3) than for *OK107>GFP* animals (64514.8 µm^3±^5186.3) at 1 day PE (Fig. 1E). However, the overall structure and proportionality of the lobes appeared intact. No obvious large-scale misrouting or deletions were observed, and upon close examination of image Z-stacks, the expanded lobes did not invade the opposite brain hemisphere. In addition to enlarged neural projections, the cell bodies of the Kenyon cells were also increased in size (Fig. 2B). Cell size was quantified by measuring the area of an optical slice through the center of the cell body using ImageJ software. Five cells each from >18 samples per genotype were measured, and then all cell measurements were averaged for each genotype. At 1 day PE, the average individual Kenyon cell area for *OK107>GFP, Rheb* animals (21.69 µm^2±^5.95) was nearly twice that of control *OK107>GFP* animals (11.15 µm^2±^3.12; Fig. 2E). However, when we compared DAPI staining within the Kenyon cells of *OK107>GFP, Rheb* animals, we did not observe an increase in size or intensity of staining as compared to control animals, indicating a lack of DNA endoreplication (Fig. 2B). We also measured the total volume of the Kenyon cell body cluster within a brain hemisphere by outlining the area of each slice containing cell bodies, then using ImageJ to estimate the entire volume of the cell body cluster across all the image stacks. Cell body cluster volume was significantly larger for *OK107>GFP, Rheb* animals (96165.67 µm^3±^38068.65) than for *OK107>GFP* animals (31952.04 µm^3±^17296.1) at 1 day PE (Fig. 2F).

### Continuous Rheb overexpression in the mushroom bodies of aged animals

We also sought to examine the effects of prolonged Rheb overexpression in the neurons of aged animals. We examined the mushroom bodies of *OK107>GFP* animals and *OK107>GFP, Rheb* animals that were collected 1 day PE and housed for 3 weeks before dissection. As seen in Figure 1, the mushroom body axonal lobes of *OK107>GFP, Rheb* animals at 21 days PE appeared grossly enlarged compared to control animals of the same age (Fig. 1C, D). The average neuropil volume of *OK107>GFP, Rheb* animals (220499.3 µm^3±^22516.4) was significantly larger than that of *OK107>GFP* animals (89337.2 µm^3±^12487.1) of the same age (Fig. 1E). Additionally, GFP staining of *OK107>GFP, Rheb* animals at 21 days PE appeared punctuate in some places and diffuse in others. Both the axonal lobes and the cell body clusters were less compact and defined, and also had more irregular and less defined GFP staining than in control animals of the same age (Fig. 2D). The average Kenyon cell body area for 21 days PE *OK107>GFP, Rheb* animals (31.72 µm^2±^11.8) was significantly larger than in control animals at either 1 or 21 days PE (11.15 µm^2±^3.22; 9.05 µm^2±^3.12; Fig. 2E). Additionally, the average Kenyon cell body area in aged animals overexpressing Rheb was significantly larger than in young animals overexpressing Rheb (21.69 µm^2±^5.95), indicating that Kenyon cell size continued to increase with continuous Rheb overexpression in aging animals (Fig. 2E). The average Kenyon cell cluster volume for cells overexpressing Rheb at 21 days PE (108332.6 µm^3±^69221.92) was significantly larger than in control animals at 21 days PE (23748.28 µm^3±^14688.06); however, the cluster volume did not significantly increase for *OK107>GFP, Rheb* animals between 1 and 21 days PE (Fig. 2F).

### Rheb overexpression in the mushroom bodies causes a memory deficiency

**Figure 3:**
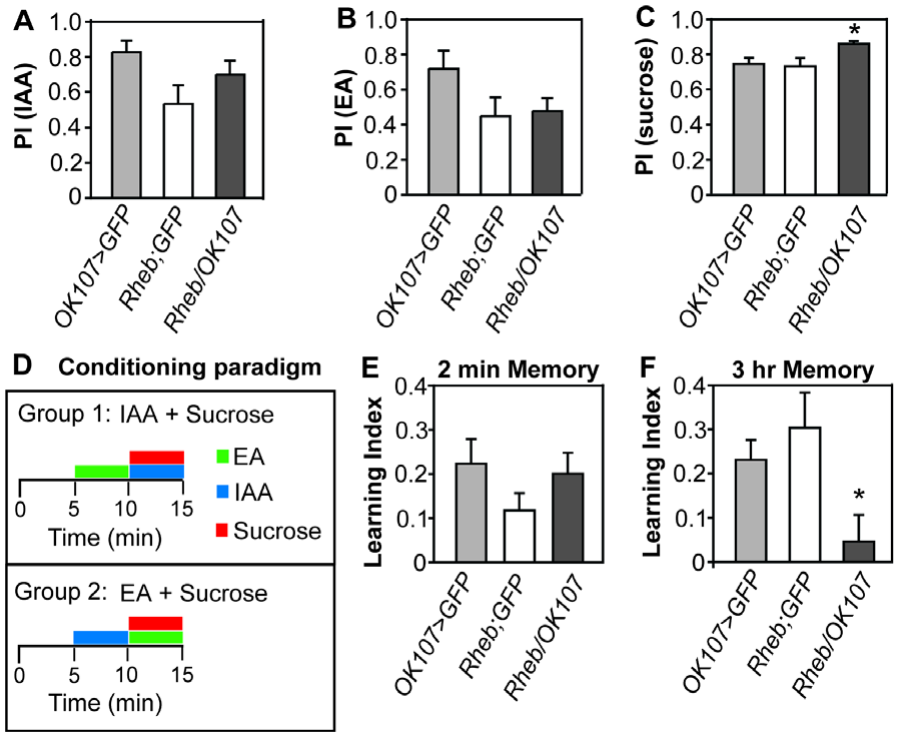
Overexpression of Rheb in the mushroom bodies decreases memory for sucrose reward. (A) Preference for the iso-amyl alcohol odor or (B) ethyl acetate odor did not significantly differ between flies in which Rheb was overexpressed in the mushroom body and heterozygous control strains (N = 8/strain, ANOVA, p>0.05 for each odor). (C) Overexpression of Rheb in the mushroom body increased sensitivity for sucrose in flies food deprived for 24 hrs (N = 16/strain, ANOVA, p = 0.02). (D) Flies food deprived for 20 hrs were given 5 min of an attractive odor with filter paper soaked in water (sucrose−), then 5 min with a different attractive odor with filter paper soaked in 2 M sucrose solution (sucrose+). They were then flipped into an empty vial and left for 2 min or 3 hr before given the choice between the two attractive odors. Preference for the sucrose+ odor was calculated by subtracting the number of flies that move toward the sucrose− odor from the number of flies moving toward the sucrose+ odor and dividing this number by the total number of flies. A reciprocal group with the opposite odor paired with sucrose was trained at the same time and a learning index was calculated by averaging the two preference indexes from the reciprocal groups. (E) Overexpression of Rheb in the mushroom body did not affect memory 2 min after training (N = 16/strain, ANOVA, p>0.05). (F) Overexpression of Rheb in the mushroom body affects memory 3 hrs after training (N = 16/strain, ANOVA, p = 0.02). Error bars represent SE.

The dramatic increase in mushroom body lobe size caused by overexpression of Rheb prompted us to investigate whether a behavioral phenotype was also present. The mushroom body plays an important role in integration of information and decision-making in the fly [29], [35], [36]. It has been implicated in memory for shock, sucrose, ethanol, and courtship [25], [27], [37]. The relatively stable nature of appetitive sucrose memory is an enticing model to study memory in the mushroom body due to the simplicity of the assay and the stability of the memory after a single training session [38]–[40]. Thus, we chose to test immediate memory (2 min after training) and 3 hr memory (3 hrs after training) using a sucrose reward paired with one of two odors. Neither of the control strains nor the flies overexpressing Rheb showed a preference for either of the two odors used (iso-amyl alcohol or ethyl acetate) when not paired with sucrose (Fig. 3A, B). Overexpression of Rheb did result in an increased sensitivity for sucrose after starvation as compared to controls potentially due to expression in the IPCs (Fig. 3C). Immediately after conditioning, control flies and *OK107>GFP, Rheb* flies had a similar learning index (Fig. 3D, E). However, 3 hours after conditioning, controls had a significantly higher learning index than *OK107>GFP, Rheb* flies (Fig. 3F), indicating that flies overexpressing Rheb in the mushroom bodies had normal immediate memory formation, but were deficient in retaining those memories.

### Rheb overexpression in the IPCs

**Figure 4:**
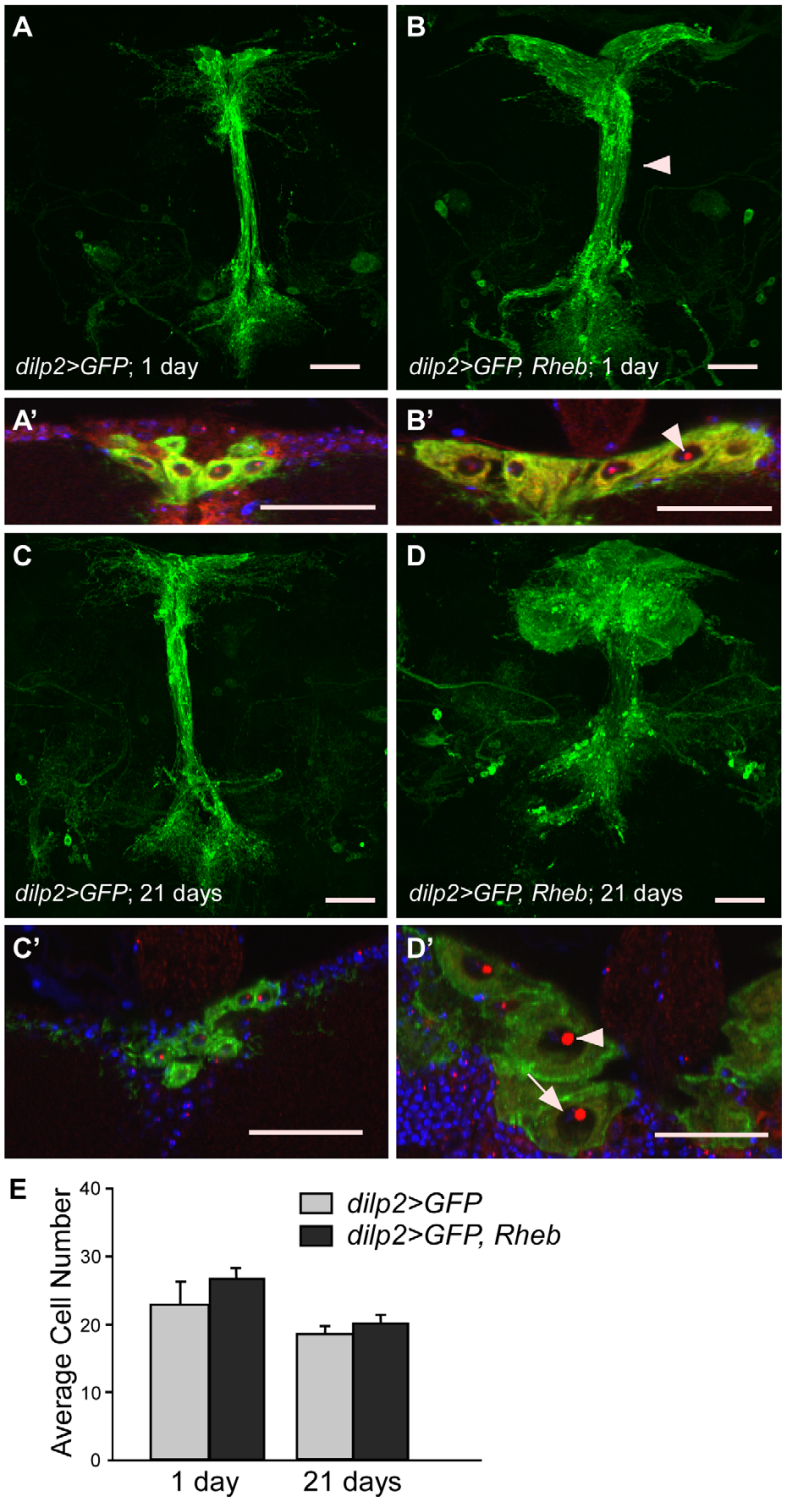
Overexpression of Rheb in the Insulin Producing Cells (IPCs) increases cell size but not cell number. (A) A Z-stack projection of *dilp2*-driven CD8::GFP in 1 day PE IPC bodies and neural projections. (A′) Close up of IPC bodies; DNA stained with DAPI (blue), and anti-fibrillarin marks the nucleoli (red). (B-B′) Rheb overexpression in 1 day PE IPCs using the *dilp2* driver causes enlarged neural projections (arrowhead, B) and cell bodies (B′). Arrowhead in B′ indicates the enlarged nucleolus. (C-C′) IPC bodies and neural projections at 21 days PE. (D-D′) Overexpression of Rheb in 21 days PE IPCs results in greatly enlarged neural projections (D) and cell bodies (D′). Arrowhead indicates the large nucleolus; arrow indicates DAPI staining in the nucleus. All scale bars are 30 µm. (E) Quantification of IPCs marked with *dilp2*-driven CD8::GFP. There is no significant difference between numbers of control and Rheb overexpressing IPCs at either 1 or 21 days PE (N = 11, ANOVA, p>0.05 for all comparisons). Error bars represent SE.

As noted previously, the *OK107* driver also expresses in the insulin-producing cells (IPCs) of the *Drosophila* brain (Fig. 1A). The IPCs are a set of neurosecretory cells that lie along the median of the *pars intercerebralis* and secrete insulin-like peptides that control growth and metabolism [41]–[44]. When comparing the mushroom bodies of *OK107>GFP, Rheb* animals to control *(OK107>GFP)* animals, we noticed that the IPCs were also expanded in size. To examine their altered morphology in more detail without overlap of the mushroom bodies, we used the *dilp2-Gal4* driver to target Rheb overexpression specifically to the IPCs. As seen in Figure 4, overexpression of Rheb visibly increased cell body size in the IPCs of 1 day PE animals. Nucleolus size (as seen by fibrillarin staining) was also increased, indicating upregulation of ribosome biosynthesis (Fig. 4B′, arrowhead). Additionally, the diameter of the individual neurites projecting from the cell bodies appeared larger, as did the entire axon bundle (Fig. 4B, arrowhead). *dilp2-Gal4>Rheb* animals did not appear to have enlarged mushroom bodies compared to controls, indicating that the size increases seen previously with the *OK107* driver were cell autonomous (not shown). Similarly to the Kenyon cells of the mushroom bodies, IPCs overexpressing Rheb continued to increase in size with continued Rheb overexpression over time. The IPC bodies of 21 days PE *dilp2>GFP, Rheb* animals appeared dramatically enlarged with huge nucleoli (Fig. 4D′, arrowhead). Again, as in the Kenyon cells, DAPI staining of DNA was not visibly increased (Fig. 4D′, arrow), signifying the *UAS-Rheb* transgene does not cause DNA endoreplication.

Because of the IPCs' irregular shape, we were unable to quantify cell size between genotypes or ages. However, because of the relatively low number of IPCs, we were able to evaluate cell number to determine if extra cell divisions had occurred. Quantification of the average IPC number per animal resulted in no significant difference between *dilp2>GFP, Rheb* animals (26.82^±^4.51) and control *dilp2>GFP* animals (23^±^3.38) at 1 day PE (Fig. 4E). Cell number was also not significantly different between *dilp2>GFP, Rheb* animals (20.1^±^4.43) and *dilp2>GFP* animals at 21 days PE (18.5^±^3.78; Fig. 4E). Animals were also fed 5-ethynyl-2′-deoxyuridine (Click-iT™ EdU) to test for EdU incorporation during S-phase. Larvae were fed EdU during their entire larval life and examined at 1 day PE; adults were also fed EdU during the first 10 days PE and examined on day 10 PE. No EdU incorporation was seen in the IPCs of either case (data not shown), further substantiating the lack of extra cell divisions and endoreplication in the IPCs of *dilp2>GFP, Rheb* animals.

## Discussion

To explore how Rheb overexpression alters neuron growth and morphology, and ultimately how the behavior of the whole organism is affected, we overexpressed Rheb in two specific neuronal subsets of the central brain of *D. melanogaster*. Selective overexpression of Rheb in two different subsets of central brain neurons induced both enlarged cell bodies and projections, while continuous Rheb overexpression over time enhanced this phenotype. Rheb overexpression in the mushroom bodies also resulted in a decrease in 3 hr but not immediate odor-sucrose memory. These experiments highlight the importance of tight regulation of the upstream components of the TOR pathway for proper neural growth and function throughout development and adulthood.

Tsc-Rheb-TOR signaling is known to have a function in controlling cell body size in the nervous system. Loss of *Tsc1* or *Tsc2* in mouse pyramidal neurons results in enlarged somata, and activation of the PI3K-Akt pathway also results in larger cultured hippocampal cell bodies; this increase in size is mediated through the Tsc-Rheb-TOR pathway, as seen by upregulation of downstream effectors such as phosphorylated S6K [13], [16], [45], [46]. In flies, both Rheb overexpression and *Tsc1* null clones induce a similar enlarged cell phenotype [6], [7], [10], [21]. Additionally, neuronal cell body size increases due to Rheb overexpression have also been seen in another invertebrate, *C. elegans* [47]. The increase that we observe in Kenyon cell body size with Rheb overexpression is in line with the growth phenotypes seen in these prior studies. Although they could not be quantified due to irregular cell borders, the IPC bodies were also visibly bigger with large nucleoli. The increased size of the nucleoli indicates a rise in rRNA production and ribosome biogenesis, consistent with an increase in protein production during cell growth.

Post-mitotic growth is often due to endocycling, in which the cell undergoes growth and DNA synthesis without mitotic divisions. Cell growth of *D. melanogaster* salivary gland cells is linked to TOR pathway activation-driven endocycling [48]. However, not all cell growth is linked to DNA content; cells within an organism that have the same genome copy number show great variations in size [49]. The apparent lack of increased DNA content upon examination of DAPI staining in the Kenyon cells and IPCs indicates that the enlargement of cell size is not due to endocycling in these neurons. Additionally, we did not observe any incorporation of EdU in IPCs in animals treated as either larva or adults, signifying that S-phase did not take place in these cells post-embryonically. Therefore, cell growth in these two neuronal subsets likely takes place via TOR activation of non-endocycling coupled growth pathways that promote anabolic processes nutrient uptake, and suppress autophagy.

In addition to increases in cell body size, neurons in which the TOR pathway has been upregulated also show an increase in the size of their projections. Eliminating Tsc1 or Tsc2 activity in the mammalian brain leads to increased neurite outgrowth, formation of ectopic axons, increased size of dendritic spines, and increased dendritic branching and complexity [13], [16], [45], [50], [51]. We also observed an increase in the overall size of the axon bundles of both the mushroom body neurons and the IPCs overexpressing Rheb. Because of the simple projection pattern, low number of axons and high GFP expression in the membrane of the IPCs, we were able to observe an increase in axon diameter in the IPCs overexpressing Rheb. However we did not observe grossly misrouted axons. This is consistent with results from other studies in *D. melanogaster*; overexpression of Rheb in the motoneuron of the neuromuscular junction results in increased synapse size but no misrouting [21] and PI3K overexpression in cholinergic central neurons causes an increase in axon diameter [52]. Conversely, misrouting has been observed in photoreceptor cells lacking Tsc1 [21]. Additionally, although both the mushroom body neurons and the IPCs showed dramatic size increases due to Rheb overexpression, overexpression of Rheb or deletion of *Tsc1* in other classes of neurons, such as the adult-specific neurons of the thoracic ganglia, did not (unpublished data). These data indicate that the effects of Rheb overexpression or *Tsc1* null clones in various neuron types may be context-dependent.

Previous studies have shown that activation of the TOR pathway can alter timing of cell divisions. Fingar et al showed that the S6K1 and eIF4E pathways downstream of mTOR promote cell cycle progression [53], while activation of PI3K-TOR in *Xenopus* embryos results in abnormally rapid cell cycles after the midblastula transition [54]. In *D. melanogaster*, it has been reported that overexpression of Rheb can lead to cell cycle activation in photoreceptor cells and S2 cells but not wing cells, indicating that effects on cell cycle of the TSC-Rheb-TOR pathway are cell-type dependent. Since the *dilp2-Gal4* driver is activated post-mitotically in the IPCs [44], [55], any additional IPCs would have to come from cell cycle re-entry. We did not find any evidence of cell cycle re-entry by the IPCs, either by cell number comparison or EdU staining in *dilp2>GFP, Rheb* flies. This could be due to the lapse of DNA repair in terminally differentiated neurons that can lead to cell death when mitosis is triggered [56]. This lack of cell divisions is also seen when an input upstream of the TOR pathway is activated; expression of PI3K in a group of cholinergic central neurons causes an increase in cell body size and number of synapses, but not an increase in cell number [52].

A previous study showed that flies with continuous ubiquitous overexpression of Rheb for 30 days post-eclosion show a decrease in geotaxis escape behavior as compared to either control animals of the same age or younger flies (5 days PE) also overexpressing Rheb in all tissues [57]. Additionally, prolonged expression of Rheb in fly photoreceptors results in degeneration of rhabdomeres due to the loss of autophagy [10]. Based on these data, we decided to monitor neuron morphology after 21 days of continuous Rheb expression in adult flies. The significant size increase in the Kenyon cell bodies relative to controls or to 1 day PE Kenyon cells overexpressing Rheb indicates that cell growth, and therefore nutrient uptake, continues. Although cell size could not be measured in the IPCs, we were able to observe an obvious increase in cell body and nucleolus size, indicating that macromolecule synthesis and ribosome biogenesis continue to increase as Rheb overexpression continues. The appearance of the cell bodies and neurite projections (bulging membranes and punctate, patchy GFP expression) in these animals at 21 days PE points to declining neuron health and is consistent with reports of Rheb-triggered neurodegeneration via inhibition of autophagy [10], [11]. Interestingly, increased phosphorylation of the human *TSC2* gene which inhibits its function in repressing TOR activity, has been found in the frontal cortex of Alzheimer's and Parkinson's disease patients [58]. These studies, in conjunction with our data, indicate that damage to neurons may accumulate with continuous TOR pathway upregulation in TSC patients.

Given the dramatic cell enlargement seen with Rheb overexpression in the mushroom bodies, we sought to examine whether this alteration would affect learning and memory. Because of the dramatic phenotype seen with Rheb overexpression, we expected a correspondingly dramatic behavioral phenotype. As expected, the capacity to form lasting memories of an odor-evoked stimulus was depressed; however, memory tested immediately after training was normal. This suggests that increased mushroom body size and/or altered cell metabolism is more detrimental to longer-lasting memories than immediate memories.

Olfactory information in *Drosophila* enters the brain through olfactory receptor neurons that connect to projection neurons (PNs) within the antennal lobe. The PNs then innervate the Kenyon cells of the mushroom body (reviewed in [29]). Memory traces have been found to occur in the PNs, the dorsal paired medial neurons (DPMs) that innervate the mushroom body lobes, and the mushroom bodies themselves [59], [60]. Information about appetitive stimuli travels through octopaminergic neurons [61] and dopaminergic PAM neurons [62], while the Neuropeptide F neurons [63] and neurons of the protocerebral posterior lateral 1 dopaminergic cluster [64] are also involved in reward memory formation. Although the circuitry involved in appetitive memory is not well understood, at least two memory traces for odor-sugar association may exist: one in the first-order interneurons (PNs) and another in the second-order interneurons (Kenyon cells) of the olfactory system in *Drosophila* [59]. The normal 2 min sucrose memory in flies with Rheb overexpression in the mushroom body suggests a mushroom-body independent memory trace, which we hypothesize may be localized to the projection neurons. Intriguingly, the projection neurons have been reported to support a 3 hr memory trace in flies [59]. Our data suggest that the memory trace in the projection neurons may be dependent on a normally functioning mushroom body. However, we cannot rule out the possibility that other neuronal structures can compensate for the defects seen in mushroom body morphology, potentially rewiring some or all of the memory circuit.

The rapid decay of reward memory seen with Rheb overexpression suggests that altered mushroom body morphology and/or Rheb signaling in the mushroom body affects consolidation or retention of memory. This is reminiscent of the phenotype observed in mutants of the Drosophila homolog of pituitary adenylate cyclase-activating peptide (PACAP) called *amnesiac* (*amn*) [65]. *amn* is required in the dorsal paired medial (DPM) neurons for consolidation of 3 hr sucrose reward memory [65]. Furthermore, DPM neuron output to the mushroom body α′β′ lobe neurons is required for memory consolidation suggesting that activity in α′β′ neurons establishes a recurrent α′β′ neuron-DPM neuron loop that is necessary for consolidation of memory [66]. Recent data suggests that the DPM neurons form a time-extended and broad olfactory cellular memory trace after appetitive conditioning [60]. Since the *OK107* driver is not expressed in the DPM neurons [33], [34], we hypothesize that the grossly enlarged morphology of the mushroom body axonal lobes is either preventing formation of a normal memory trace in the mushroom body, or preventing proper synaptic connectivity with the DPM neurons, resulting in lack of consolidation.

Intriguingly, appetitive memory decay also occurs more quickly when a non-nutritive sugar is used, and this decay correlates with the duration and breadth of the DPM neuron memory trace [60], [67], [68]. Thus, another possibility is that Rheb overexpression in the mushroom body and/or IPCs results in inability to process sugar. We believe however, that the normal body development and size (data not shown), along with the increased sucrose sensitivity in *OK107>Rheb* flies, makes this possibility less likely.

Disruption of neuronal morphology and function has also been seen in vertebrate models. Deletion of *Pten*, an upstream negative regulator of TOR, results in mice with ectopic neural processes and increased dendritic spine density. These mice also show decreased learning ability [69]. In addition to growth and misrouting phenotypes, suppression of TSC1 or TSC2 function has been shown to affect neuronal polarity, neurotransmitter receptor expression, neuron hyperexcitability and reduced synaptic plasticity [70]–[73]. Indeed, Eker rats heterozygous for a *Tsc2* mutation show reduced long-term potentiation and long-term depression in hippocampal cells, indicating that the synapses may have a reduced ability for activity-dependent synaptic modification, a necessary part of memory formation [73].

The role of the Tsc-Rheb-TOR signaling pathway in determining neuron size, growth, projection pattern and function is complex, and depends on cell type, species, age of the organism, and environmental conditions. However, studies in both vertebrates and invertebrates are yielding clues as to the specific effects tuberous sclerosis has on the various cell types of the nervous system, both in organization and in functional capacity. Our results add to this growing body of work, establishing another model for exploration of the effects of upstream regulators of TOR on neuron morphology and function.
